# Microbes in nature are limited by carbon and energy: the starving-survival lifestyle in soil and consequences for estimating microbial rates

**DOI:** 10.3389/fmicb.2013.00324

**Published:** 2013-11-12

**Authors:** John E. Hobbie, Erik A. Hobbie

**Affiliations:** ^1^The Ecosystems Center, Marine Biological LaboratoryWoods Hole, MA, USA; ^2^Earth Systems Research Center, University of New HampshireDurham, NH, USA

**Keywords:** microbes, soil, water, activity, labeled substrate, amino acids, sugars

## Abstract

Understanding microbial transformations in soils is important for predicting future carbon sequestration and nutrient cycling. This review questions some methods of assessing one key microbial process, the uptake of labile organic compounds. First, soil microbes have a starving-survival life style of dormancy, arrested activity, and low activity. Yet they are very abundant and remain poised to completely take up all substrates that become available. As a result, dilution assays with the addition of labeled substrates cannot be used. When labeled substrates are transformed into ^14^CO_2_, the first part of the biphasic release follows metabolic rules and is not affected by the environment. As a consequence, when identical amounts of isotopically substrates are added to soils from different climate zones, the same percentage of the substrate is respired and the same half-life of the respired ^14^CO_2_ from the labeled substrate is estimated. Second, when soils are sampled by a variety of methods from taking 10 cm diameter cores to millimeter-scale dialysis chambers, amino acids (and other organic compounds) appear to be released by the severing of fine roots and mycorrhizal networks as well as from pressing or centrifuging treatments. As a result of disturbance as well as of natural root release, concentrations of individual amino acids of ~10 μM are measured. This contrasts with concentrations of a few nanomolar found in aquatic systems and raises questions about possible differences in the bacterial strategy between aquatic and soil ecosystems. The small size of the hyphae (2–10 μm diameter) and of the fine roots (0.2–2 mm diameter), make it very difficult to sample any volume of soil without introducing artifacts. Third, when micromolar amounts of labeled amino acids are added to soil, some of the isotope enters plant roots. This may be an artifact of the high micromolar concentrations applied.

## INTRODUCTION

A major goal of microbial ecology is to connect the processes or functions occurring in aquatic and terrestrial ecosystems with the microbes present in those systems. A major function of heterotrophic bacteria and fungi in nature is to break down large organic molecules, transport low molecular weight (LMW) compounds into microbial cells, and use a portion of the LMW compounds for respiration and growth. There is agreement among authors that the concentration and supply rate of bioavailable organic material determine the growth of heterotrophic microbes (Coleman et al., 2004; Egli, 2010; Inselsbacher and Näsholm, 2012). These heterotrophic microbes and the methods used to study them are the subject of this review. In particular, this review deals with methods of measuring microbial rates of use of LMW compounds in soil and compares these with similar methods developed for aquatic ecosystems. This comparison between terrestrial and aquatic ecosystems is based on the premise that metabolic and stoichiometric constraints on microbial metabolism are very similar across ecosystems (Sinsabaugh et al., 2013). Three types of commonly used soil methods appear to produce doubtful results and are described in Section “Observations and Concerns.”

### AQUATIC MICROBES: MEASURES IN THE PLANKTON

For some questions, planktonic systems are easier to study than soil systems; they contain fewer microbes and lack the structure caused by roots and soil particles. The ideal method for measuring heterotrophic processing of LMW compounds in natural systems is to measure the concentration, uptake rate, and respiration rate of the compounds of interest. Uptake of LMW compounds accounts for the majority of carbon used by heterotrophic bacteria. For example, Kirchman (2003) reported that the flux of free amino acids or of glucose alone can support most or all of the bacterial growth. With high pressure liquid chromatography, individual LMW compounds such as sugars and amino acids can be measured to concentrations as low as a few nanomoles per liter. Uptake and respiration rates in aquatic systems are easily measured by adding ^14^C- or ^13^C-labeled compounds to samples from nature and incubating for a few hours. Subsequently, the quantity incorporated into microbes caught on filters (as fine as 0.2 μm pore size) and released as CO_2_ is measured. Turnover times of individual LMW compounds may be calculated as the concentration divided by the rate of uptake; when actual incorporation into protein is measured, the bacterial growth rate may be estimated (Fuhrman and Azam, 1982; Kirchman, 2012).

Bacterial abundance is close to 10^6^ ml^-^^1^ in the plankton of lakes, estuaries, and oceans. Growth rates vary greatly but likely average around 0.2 day^-^^1^ (Pomeroy et al., 2007); in oligotrophic waters rates are very slow, less than 0.01 day^-^^1^, while in rich estuaries they may be as high as 1 day^-^^1^ (Crump et al., 2013). Concentrations of sugars and amino acids are very low, with individual free amino acids ranging from <1 to 20 nM and the total concentrations of amino acids usually <100 nM (Fuhrman, 1987; Kirchman, 2012); the concentration of glucose and other free neutral sugars is <5 nM (Kirchman, 2000). Microbes are adapted to the extremely low concentrations of amino acids and sugars. In fact, bacteria in aquatic systems likely control the concentration of LMW compounds (Nissen et al., 1984). The turnover time of minutes to hours is rapid enough to result in high fluxes. In a eutrophic estuary in southeastern United States, turnover times of amino acids were as low as 0.7 h at summer temperatures and 206 h at winter temperatures (Crawford et al., 1974).

### SOIL BACTERIA

Bacterial abundance is close to 10^9^ per gram or several magnitudes greater in soils than in plankton (Whitman et al., 1998). When the total respiration of unmodified soil is used to calculate microbial growth, generation times are estimated at 120–180 days (Coleman et al., 2004); these authors attributed the slow rate to the extreme limitations of available carbon compounds. Faster turnover times are estimated from the microbial incorporation of labeled leucine or thymidine to estimate bacterial growth. With these methods, generation times ranging mostly from 2 to 13 days have been found (Bååth, 1998; Kirchman, 2000; Rousk and Bååth, 2011). Thus, bacteria are abundant and active in soils. Yet, in contrast to aquatic microbes these organisms apparently do not hold the concentrations of LMW compounds at a very low level. For example, concentrations of free amino acids found in soils is high and rather constant. Total free amino acids in 40 soils from around the globe had a mean concentration of 23 ± 5 μM (SE) or nearly 1000 times the concentration in natural waters (Jones et al., 2009).

It should be noted that fungi are also abundant in soils but rare in the plankton and anaerobic salt marsh soils. In terrestrial soils, fungal biomass can be close to that of bacterial biomass (Hobbie and Hobbie, 2012) and fungal turnover is tens to several hundred days (Rousk and Bååth, 2011). In this review, measures of microbial uptake, respiration, and growth are considered to include both bacteria and fungi.

### OBSERVATIONS AND CONCERNS

The methods that we are concerned about fall into three categories. Each is first described in this section along with the concerns for the quality of the method. In the next sections, the explanations for the concerns and of the characteristics of microbes in nature are discussed:

(1) There is a very rapid microbial uptake of any and all added LMW compounds in experiments. The concern is the fact that starving bacteria in soil exist at low levels of activity yet can take up any added and readily available substrate; this rapid removal is a result of the high numbers of bacteria and does not represent the natural occurring rates.
(2) High concentrations of LMW compounds are measured in soils while extremely low concentrations are present in planktonic systems. Given the presumed ability of planktonic bacteria to draw down available concentrations to nanomolar concentrations, why then do soil bacteria exist in a medium where the concentration of LMW compounds are at micromolar levels? The seeming disparity in concentrations is probably caused in part by mixing of the soil or severe disturbance of fine roots and mycorrhizal networks during soil preparation that release LMW compounds previously unavailable to microbes.
(3) High rates of release of labeled CO_2_ are measured in experiments in which labeled LMW compounds are added. These rates are interpreted to be equivalent to respiration of LMW compounds in nature and the release is used to estimate a half-life of these compounds in the soil, which is usually a few hours. However, once the added substrate is taken up by microbes, then the release rate is controlled by the internal metabolic pathways and not by external environmental conditions. More realistic turnover times, measured in days, are estimated from the ^14^C held in microbial biomass and not released in the first burst of high respiration.

## THE STARVING-SURVIVAL LIFESTYLE

In his 1997 book, titled *Bacteria in Oligotrophic Environments: Starvation-Survival Lifestyles*, Richard Morita reviewed the marine and terrestrial literature on microbial ecology and concluded that most of the biosphere is oligotrophic – and that this should be considered the normal state of most environments. He exempted rich coastal regions of the oceans, eutrophic lakes, and the rhizosphere (see also a discussion of the same theme in *Fundamentals of Soil Ecology*; Coleman et al., 2004). Furthermore, he also believed that the microbial response to oligotrophy, a starvation-survival lifestyle, is the normal physiological state of microorganisms in nature (Morita, 1988, 1997). At any moment, some microbes are active, some are in a state of arrested activity, and some are in a dormant state. Even when in a low activity phase, microbes maintain the biochemical machinery necessary to use any exogenous substrate. A similar concept concludes that the soil microbial community is largely inactive (Kuzyakov et al., 2009).

**FIGURE 1:**
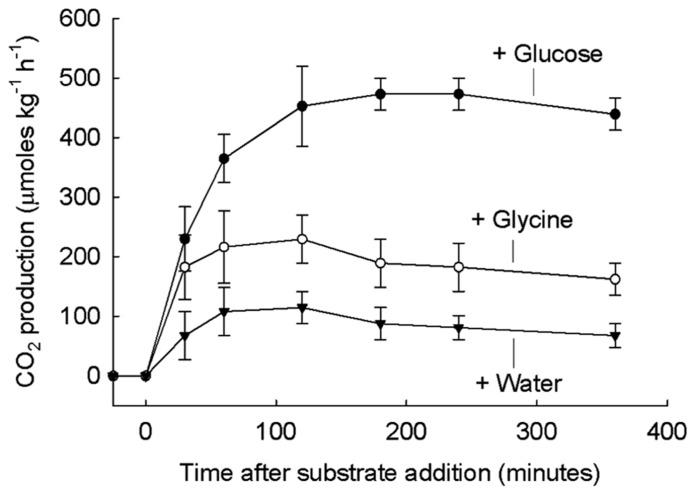
**Soil respiration after addition of glucose, glycine, or water.** 50 mM glucose (top), 50 mM glycine (middle), or rainwater (bottom) added to grassland soil at time 0. The basal respiration value of 209 μmol CO_2_ kg^-^^1^ h^-^^1^ has been subtracted from all treatments. From Jones and Murphy (2007) with permission.

A major aspect of the starving-survival lifestyle is that large numbers of microbes with low activity are poised to respond quickly to added substrate (White, 1995). This aspect of the survival lifestyle is very important to consider when the response (such as CO_2_ release) of large numbers of activated soil microbes is measured. This response is less important for measurements in aquatic systems where there are many-fold fewer microbes. Soil microbes are physiologically adapted to respond rapidly. When soil was held in the laboratory for many months (Brookes et al., 1983; De Nobili et al., 2001) microbes maintained ATP and an adenylate charge ratio (AEC) of 0.8, typical of exponentially growing microbes *in vitro*. The AEC is defined as the quantity (ATP + 0.5 ADP)/(ATP + ADP + AMP). The rapidity of the respiration response of soil microbes, just a few minutes, to very high micromolar amounts of added glucose and glycine is illustrated in **Figure 1** (Jones and Murphy, 2007).

After an amino acid or a sugar is taken up by the microbial cell, the subsequent release of ^14^ CO_2_ is controlled by the metabolic pathways within the cell (details in Hobbie and Hobbie, 2012). Because the fundamental pathway leading to respiration of a given amino acid is generally the same for most microbes, the percentage of substrate carbon converted into CO_2_ for a given amino acid is the same for many microbial communities and is likely a function of the length of the catabolic pathway (Hobbie and Crawford, 1969). For example, the proportion of amino acid that was respired after addition to a freshwater pond and an estuary for glutamic acid and aspartic acid were always 50–60% while those of basic amino acids, such as leucine at 14% and lysine at 12–14%, were low (Hobbie and Crawford, 1969). These percentages for the individual amino acid taken up into microbial cells were confirmed in a eutrophic lake (Wright, 1974). It is now known that these percentages are the same for microbes in productive freshwaters and estuaries but the percentage of leucine respired increased up to 82% in ultraoligotrophic ocean waters (Alonso-Sàez et al., 2007). Data from the few soil respiration measures are very similar to the aquatic values (Rousk et al., 2011; Hobbie and Hobbie, 2012). It is important to note that in aquatic systems the percent respired is of the amount of the isotope taken up into the cell while in soil all the added substrate is taken up by microbes so the percent respired is of the amount added in the experiment. Thus 45% and 20% of added glutamic acid and lysine, respectively, were respired in a Spanish farm soil (Vinolas et al., 2001) and 50% of added aspartic acid was respired in tundra soils in Alaska (Nordin et al., 2004). In a careful comparison of 40 soils from around the world (polar, tropic, and temperate zones, cultivated, grassland, and forest soils), a mix of 15 amino acids added to soil produced the respiration of exactly the same percentage of amino acid carbon (of that added) in all the soils (Jones et al., 2009).

A similar conclusion to the above is given, in somewhat different words, in a description of the biphasic pattern of ^14^CO_2_ evolution in mineralization studies (Oburger and Jones, 2009). They describe a rapid mineralization phase (phase 1) that is largely independent of the experimental conditions (our metabolic control). A subsequent second phase (phase 2) had a much longer release time for substrate and was significantly affected by incubation conditions. There is a question, however, about what organic compound the ^14^C is in during the long release period.

## LMW COMPOUNDS IN OCEANS, LAKES, AND SOILS: CONCENTRATIONS AND SOURCES

### SUGARS, ACETATE, AND AMINO ACIDS IN AQUATIC AND TERRESTRIAL ECOSYSTEMS

The concentrations of sugars, such as glucose and other free neutral sugars, is extremely low in planktonic aquatic systems. In general, these systems contained <5 nM for individual sugars (Kirchman, 2000) while 2–15 nM of glucose were measured in surface waters of the Gulf of Mexico (Skoog et al., 1999). Somewhat higher concentrations, 20–60 nM, have been found in the equatorial Pacific (Rich et al., 1996).

Sugar concentrations in soils appear to be much higher than in aquatic systems, although the variety of methods used makes exact comparisons difficult. For example, 54 μM glucose was reported in an agricultural soil in North Wales, with sugars extracted by centrifugation of soil samples at 4000 *g* (Hill et al., 2008). Sugars extracted from soil of temperate forest plantations with 0.25 M sulfuric acid for 16 h averaged 244 mg kg^-^^1^ soil (equivalent to 4.5 mM, assuming 0.3 g water g^-^^1^ soil; Johnson and Pregitzer, 2007), whereas concentrations from water leachates of only 2.4 μM sugars (~30% glucose) were reported from an agricultural soil (Fischer et al., 2007).

A similar picture is found for dissolved free amino acids (**Tables 1** and **2**); the concentration in the upper waters of aquatic systems is a few nanomolar while soil water concentrations are in the micromolar range. As seen in **Table 1**, the concentration is higher in the more eutrophic waters (coastal, estuarine) than in open ocean waters.

**Table 1:** Concentrations of dissolved amino acids measured in lakes, estuaries, and oceans.

Location	Amino acid concentration (nM)	Notes
Ocean^a^	0.1–50	Individual amino acids
Coastal Ocean (New York Bight)^b^	1–15	Individual amino acids
Estuary (North Carolina)^c^	300–500	12 amino acids
Productive lakes^d^	78–277^e^	Total for five sampling days

a Williams (2000).

b Fuhrman and Ferguson (1986).

c Crawford et al. (1974).

d Jørgensen (1987).

e 1200 and 1500 nM found once.

**Table 2:** Total dissolved amino acid concentration measured in soil water or soil water extracts or KCl extract.

System studied	Amino acid concentration (μM)	Notes
40 soils worldwide^a^	23 ± 5	OPA fluorometry
Boreal forest, Sweden^b^	42–106	Upper organic layers OPA fluorometry
	5–20	Lower layers OPA fluorometry
Boreal forest, Sweden^c^	133	Water extraction,
Agricultural land, Sweden^d^	0.1–12.7	Small tension lysimeters, 2–9 cm depth
Temperate grassland, Wales^e^	23–58	Total for 15 different amino acids,
		Monthly for 6 mo by HPLC
Pine forest, California^f^	35	Leachate of O horizon, by HPLC
Temperate forest, U.S.^g^	301	Organic horizon
	59.9	Mineral horizon

*OPA, ortho-phthaldialdehyde.*

a Polar, temperate, tropical, agriculture, non-agriculture (Jones et al., 2009).

b van Hees et al. (2008).

c Inselsbacher and Näsholm (2012). 0.3 g water assumed per g soil.

d Jämtgård (2010).

e Jones et al. (2005b).

f Yu et al. (2002).

g Maple, ash, oak, beech, hemlock. Soil extracted with KCl immediately after collection, and amino acids assessed by ninhydrin method. For calculation of concentration, 0.3 g water assumed per g soil and published bulk density used (Gallet-Budynek et al., 2009).

If, as widely believed, the microbes in soil are carbon and energy limited (Coleman et al., 2004), how can the micromolar concentrations of LMW compounds remain so high and not be removed? Are aquatic and soil microbes so fundamentally different that one group can live at concentrations of LMW compounds that are four orders of magnitude less than the other? One possibility is that the measured concentrations are correct and that the microbes have adapted to a life of plenty by developing transport systems with a relatively high *K*_m_ and that there is no competition among microbial species. A second possibility is that the diffusion of LMW compounds is very slow in soils and the amount of substrate that reaches the cells is low relative to bulk concentrations. A third possibility is that substrates are released from micropores, from severing of fine roots or the networks of mycorrhizal hyphae, or from release from loose attachment with soil particles when the sample of soil is sieved or mixed. Different ways of extracting organic substrates from soil give different concentrations; for example, Darrouzet-Nardi and Weintraub (personal communication) found that samples from arctic soils extracted with water contained five times more labile N substrates than samples collected with a lysimeter.

In fact, there is evidence for a rapid microbial response to sampling, presumably to the LMW compounds newly made available to microbes. One bit of evidence is the burst of unusually high microbial respiration when cores are first collected (G. Shaver, personal communication). Also, when grassland soil is extracted with distilled water or with K_2_SO_4_ and ^14^C-labeled amino acids and sugars are added to the solution, the organic compounds are quickly lost from solution with a maximum rate of 90% loss after 15 min (Rousk and Jones, 2010).

## ARE LMW COMPOUNDS PRODUCED FROM DESTRUCTION OF FINE ROOTS AND FUNGAL NETWORKS?

Aquatic sediments are useful for investigating this question because they are anaerobic and therefore lack mycorrhizal fungi. In beds of the salt marsh grass, *Spartina alterniflora*, dissolved organic carbon (DOC) concentrations in the anaerobic sediments are sampled by pounding in a coretube (e.g., 6.4 cm diameter), extruding the core, and extracting the water from the core by squeezing or centrifugation. A marsh in Massachusetts yielded DOC concentrations of 4–6 mM (Howes et al., 1985). Because of its importance in the anaerobic pathway of decomposition, acetate was chosen for detailed study; water extracted from a 6.4 cm diameter core by squeezing or centrifugation had acetate concentrations greater than 100 μM (Hines et al., 1994). In contrast, non-destructive collection methods found less than 10 μM acetate. These non-destructive methods either used an *in situ* Teflon sipper deployed several days before the first sampling (Hines et al., 1989) or collected water from an extruded core using a syringe and needle. In an experiment to test if the roots were the source of the acetate, all sediment was first washed from a large core and then the remaining roots were cored and destructively sampled; 75% of the acetate found in intact cores was recovered (Hines et al., 1994). Thus, in this case the roots were certainly the source of the acetate found in the core.

We now consider the evidence for production of LMW compounds from vegetated soils when bacteria, mycorrhizal fungi, and fine roots are all present. All these organisms are potential sources of the organic compounds found in soil water; it was estimated by one group that free amino acids have a concentration of 10 mM within roots (Jones and Darrah, 1994; Farrar et al., 2003; Jones et al., 2005a). Another estimate (Marschner, 1995) was that phloem sap has an amino acid concentration of 50–200 mM. In recent efforts to avoid the disruption to roots and microbes of coring or of other destructive collection and preparation manipulations, a microdialysis chamber sampled nitrogen (N) compounds (ammonium, nitrate, and amino acids) at a depth of 1 cm in the soil solution of a Swedish pine forest. This chamber, made from a semi-permeable membrane 10 mm long and 0.5 mm in diameter, received a continuous flow of deionized water at 5 μl min^-^^1^ for 30 min (Inselsbacher et al., 2011; Inselsbacher and Näsholm, 2012). After water extraction of the soil by standard methods, concentrations of free N were dominated by ammonium, up to 79% of the free N, while amino acids and nitrate made up 11 and 10%, respectively (**Table 2**). In the diffusive flux of N into the dialysis chamber, amino acids were 80% of the free N while ammonium and nitrate each contributed 10%. The authors stated that this approach measures the potential N supply rates in a system where N compounds are continually removed from solution. They suggest that this technique should give a more accurate representation of soil N supply than traditional soil sampling measures of concentration.

One potential problem with the microdialysis method as presented is that disruption of fine roots or fungal hyphae during insertion of the dialysis probe could release amino acids. However, this does not seem to be the case because E. Inselsbacher (personal communication) has a report in preparation showing that samples collected at a number of periods after the insertion of the dialysis probe into the soil show no changes in the time course of concentration.

Another type of evidence that the fungi and fine roots may be a source of the amino acids sampled during microdialysis comes from the compositional profile of amino acids sampled in ectomycorrhizal fungi and in the pine forest soils. The following data come from an experiment in which individual *Pinus sylvestris* seedlings were inoculated with four different taxa of ectomycorrhizal fungus (Finlay et al., 1988). Fungal hyphae subsequently grew from the root tip across a barrier into peat to which ^15^N-labeled ammonium was added. Labeled free amino acid pools stemming from the hyphal uptake of the label were then measured in the hyphae, the mycorrhizal root tip, the roots, and needles. Labeled nitrogen was found in all four free amino acid pools principally as glutamine/glutamic acid but significant amounts of asparagine/aspartic acid were also found (Finlay et al., 1988). When microdialysis and tension lysimeters were used to sample homogenized boreal forest soils where ectomycorrhizal fungi of pine trees were certainly abundant, the most abundant amino acid was also glutamine followed by valine, alanine, and glutamic acid (Inselsbacher et al., 2011). When the soil amino acids were sampled across a successional sequence of boreal forest plants (willow, alder, balsam poplar, white spruce, and black spruce), the amino acid pool was dominated by glutamic acid, glutamine, aspartic acid, asparagines, alanine, and histidine in every case (Werdin-Pfisterer et al., 2009). Glutamine was also the dominant amino acid in the xylem pool of ectomycorrhizal plants (Pfautsch et al., 2009). We suggest that it is possible that the microdialysis methods as well as other methods of sampling amino acids in soils, such as tension lysimeters, are measuring mostly amino acids released from fine roots and mycorrhizal hyphae. This question about the source of the amino acids in the soil water should be extensively tested.

Finally, what are implications for soil studies generally of the possibility that the breakage of fine roots and mycorrhizal networks add large amounts of LMW compounds to soils during sampling and handling? Certainly the possibility of handling error is not considered in descriptions of methods. An example is the method of studying gross N-cycling rates of aggregates in a non-rhizosphere system (Muruganandam et al., 2010) described in a chapter in Methods in Enzymology (Myrold et al., 2011). After the soil was collected it was air dried for days and then sieved. The ^15^N-labeled ammonium or nitrate was added, the soil rehydrated to 60% of water holding capacity, and the soil incubated for a week. An isotope ratio mass spectrometer was used for analysis and the net rates of change calculated from the isotope pool dilution. No mention was made about any potential errors introduced during sampling of the soil or of the effect of increased microbial activity from labile organic compounds added during sampling and preparation.

## TURNOVER OF LMW COMPOUNDS

### AQUATIC METHODS: ISOTOPE DILUTION MEASURES OF UPTAKE VELOCITY WORKS WHEN CONCENTRATION OF THE ADDED SUBSTRATE IS NEAR NATURAL LEVEL

**FIGURE 2:**
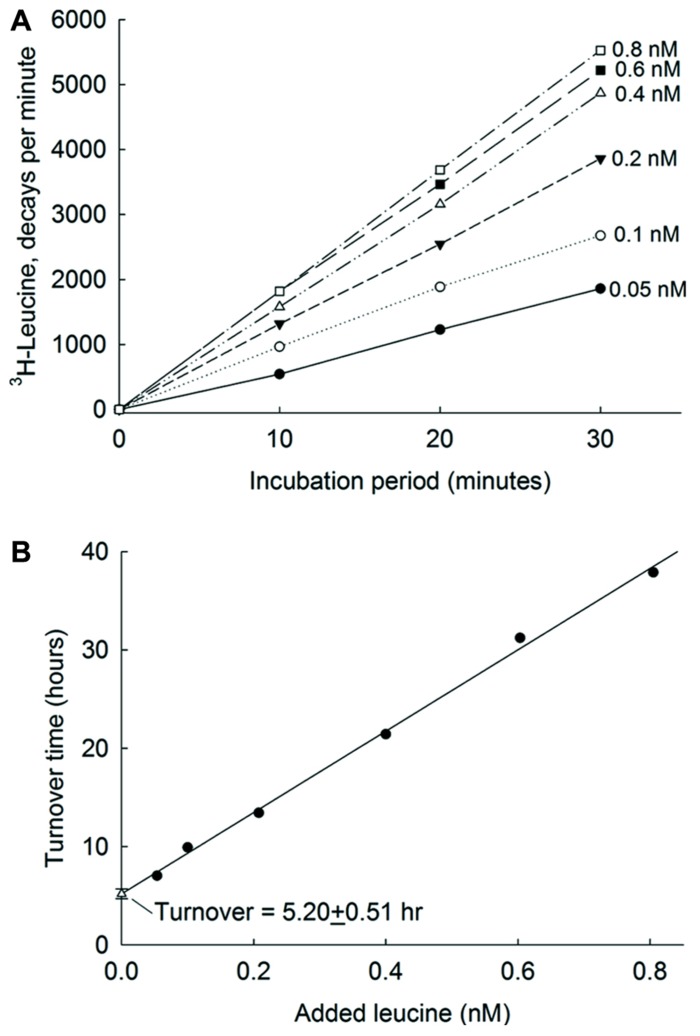
**Leucine uptake kinetics in Atlantic Ocean.** **(A)** Incorporation into bacteria of ^3^H-leucine at 6 nM concentrations. **(B)** The relationships between added amino acid concentrations and their corresponding turnover times. The error bars show single standard errors. The *Y*-axis intercept of the regression line is an estimate of turnover time at maximum bioavailable ambient concentration of amino acids. From Zubkov et al. (2008) with permission.

In planktonic systems, the turnover of organic compounds is defined as the substrate concentration (*S*) divided by the uptake velocity (*v*). This may be measured by a short-term incubation of the sample with an array of different concentrations of a labeled substrate. The incubation must take place while uptake of the bacterioplankton is directly proportional to time (as shown in **Figure 2A**). The uptake velocity is sometimes increased by including the carbon respired during the experiment. When the concentration of substrate added (*A*) is close to the concentration *S*, the uptake follows Michaelis–Menten kinetics and the experiment can be analyzed as a dilution bioassay (**Figure 2B**). When the uptake velocity, *v*, is measured at various concentrations of *A*, each result can be plotted as a turnover time for that amount (*A*) of added substrate plus an unknown natural level of substrate (*S*). The extrapolation to zero added substrate (the Y intercept) is then the turnover at the natural level of substrate (Wright and Hobbie, 1966). In ultraoligotrophic ocean waters picomolar (pM) concentrations of leucine were added for uptake studies (Zubkov et al., 2008). The kinetic analysis of water from the Atlantic Ocean incubated for 30 min (**Figure 2**) shows that leucine was present in very low concentrations (0.1–0.2 nM and that the turnover time, the intercept on the *Y* axis, was around 5 h. See also Hobbie and Hobbie (2012) for a detailed explanation of the derivation of the equations.

### SOILS METHODS: ISOTOPE DILUTION FAILS WHEN ALL ADDED SUBSTRATE IS IMMEDIATELY TAKEN UP

**FIGURE 3:**
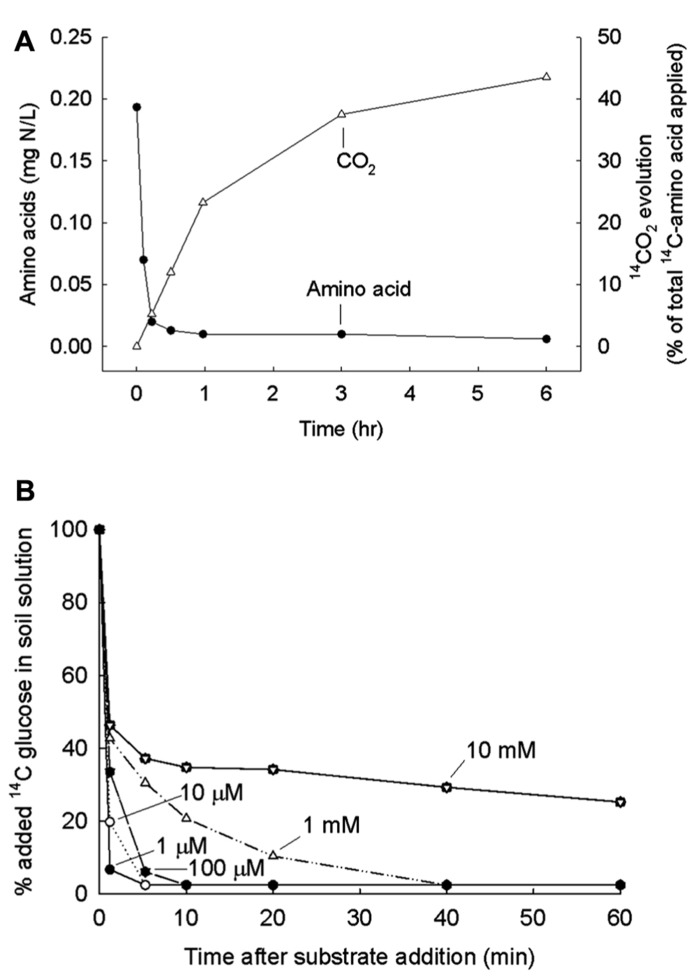
**(A)** Amount of ^14^C-amino acids remaining in soil sample after various periods of incubation. A mix of eleven labeled amino acids added and ^14^CO_2_ released also measured. From Jones et al. (2004) with permission. **(B)** Amount of ^14^C-glucose remaining in soil solution after various periods and for various concentrations in final solution. From Hill et al. (2008) with permission.

The methodology devised to examine the turnover of LMW compounds in soil is quite different from the dilution analysis that works in the plankton community. The necessity for the different methodology is shown in **Figure 3A**. In soil there are large numbers of low-activity microbes poised to respond quickly to added substrates. Because of this, uptake is immediate and complete for all of the added substrates, both amino acids and sugars; respiration of substrates to ^14^CO_2_ also begins immediately which, incidentally, is proof that microbes are involved and not inorganic processes. **Figure 3B** shows that the rate of uptake into microbes is not affected by the concentration of the added substrate, at least at concentrations below 1 mM. Isotope dilution with different quantities of added glucose or amino acid does not work when all the substrate is immediately taken up. A modification that does work is to add additional water, to use homogenization, and centrifugation to extract bacteria from the soil matrix before adding the labeled leucine, and to measure incorporation of leucine into cellular protein (Bååth, 1994, 1998; Rousk and Bååth, 2011).

### SOILS METHODS: ^14^CO_2_PRODUCTION FROM ADDED SUBSTRATE MEASURES WITHIN-CELL PROCESSES ONLY AND NOT ENVIRONMENTAL EFFECTS

**FIGURE 4:**
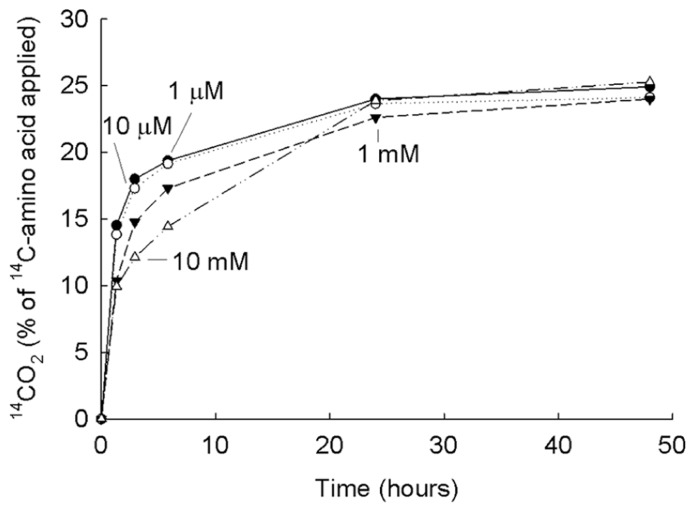
**^**14**^C-labeled glycine transformed to^**14**^CO_**2**_ over time.** The ^14^C-glycine at four concentrations (μM to mM) was added to a grassland soil (eutric cambisol) and transformation to ^14^CO_2_ measured over time. From Jones et al. (2005b) with permission.

A different way of estimating the turnover or mineralization in soil is to measure the rate of ^14^CO_2_ production after the substrate is taken up (e.g., **Figure 4** for labeled glycine). This popular method makes use of concentrations of LMW labeled compounds added to produce concentrations in the micromolar range. The result of the measurements is estimations (Hill et al., 2008) of the half-life of the total amount of ^14^CO_2_ formed from the labeled substrate; in this paper labeled glucose was added to produce a series of concentrations from 1 to 10,000 μM. The range of the calculated half-life range was 8–11 min and the assumption was made by the authors that this is equivalent to 10–1000 turnovers of the soil glucose per day. Several papers have pointed out that the ^14^CO_2_is released in two phases (Hill et al., 2008; Oburger and Jones, 2009; Glanville et al., 2012), that is, an initial rapid phase of evolution (phase 1) followed by a slower phase (phase 2).

What actually is being measured in this method? The method neither measures the actual rate of respiration nor the turnover rate of substrate in soil. It does measure the time at which half of the total respired substrate has been respired; for example, if 60% of applied glucose is ultimately respired, the time at which 30% of the glucose has been respired is estimated. It thus measures the half-life for microbial respiration of the labeled substrate. Then the assumption is made that this equals the half-life of the amino acid or sugar in the soil. The actual turnover time in the unmodified soil is not measured; we suggest that it is the turnover time for labeled substrate inside the cell that is measured. In nature, many different environmental variables would change the actual turnover time such as number of microbes, their activity, and the concentration of amino acids or sugars.

What are the characteristic of soil microbes that cause difficulty with this method of estimating substrate turnover? The first characteristic is that the microbes under energy and carbon limitation are poised to immediately take up the LMW compounds when they become available. In fact, the microbial physiology changes so that many substrates are taken up (the mixed substrate growth described by Egli, 2010). When labeled LMW compounds are added to soil, they are all immediately taken up by microbes no matter what the concentration added (from nanomolar to high micromolar levels; **Figure 3A**).

### SOIL METHODS: A GIVEN SUBSTRATE HAS THE SAME TURNOVER TIME IRRESPECTIVE OF THE ENVIRONMENT

The second characteristic, that applies to the first phase of release, is that the release rate of ^14^CO_2_ is controlled by the biochemical efficiency of pathways within the cell (see earlier discussion in the section “The Starving-Survival Lifestyle”) and not by events in the external environment. If the release rate of ^14^CO_2_ is controlled by fundamental metabolic pathways common to all heterotrophic microbes and not by the activity of the microbial community of an individual soil, then soil samples treated with the same amount of labeled amino acid will always produce similar estimates of the half-time for mineralization. This was the exact finding for 40 soils collected worldwide (Jones et al., 2009); the mean global concentration of total amino acid was 23 ± 5 μM and the half-life was 1.8 ± 0.1 h. In addition, for all 40 soils an average of 71% of the substrate (a single addition of 15 amino acids totaling 20 μM) was retained in the microbial cells and 29% was respired. Zoe Cardon and John Stark (personal communication) point out that this is exactly the summed percentage of 15 amino acids taken up and then respired in an aquatic study (Hobbie and Crawford, 1969) where each amino acid was added individually to sub-samples of a rich pond. The key factor is that microbes in both aquatic and soil systems processed the added labeled amino acids by the same fundamental biochemical pathways such as that leading to the citric acid cycle for respiration. We suggest that results from ponds and soils are so similar because the same biochemical pathways dominate microbial processing across these two environments. A similar result was found for mineralization of amino acids and sugars across a soil pH gradient; the mineralization process was the same across the gradient while the microbial communities differed dramatically (Rousk et al., 2011).

### SOILS METHODS: LONG-TERM SLOW RELEASE OF ^14^CO_2_ FROM ADDED SUBSTRATE MEASURES BREAKDOWN OF MICROBIALLY CREATED COMPOUNDS, NOT TURNOVER OF ORIGINAL SUBSTRATE IN NATURE

A different approach to measuring the turnover of LMW compounds in soil is to add low amounts of labeled compounds and to follow the release of ^14^CO_2_ for 7 days (Glanville et al., 2012). In this experiment, 31 different labeled compounds were added individually to small chambers (6.1 cm^2^) formed by pushing plastic cylinders ~2 cm into the soil of grassland in North Wales. The label (<10 nM) was added in 0.5 ml of water gently placed on the top of the soil. The ^14^CO_2_ was collected in a NaOH trap inside the chamber. The total substrate half-life was estimated as ranging from 1 to 40 days with most substrates from 5 to 30 days. This range, which is the sum of that for phase 1 and phase 2, is much longer than estimates of several hours obtained when high (μM) concentrations of labeled substrate were added and only the phase 1 half-lives reported. There is, however, a conceptual problem with phase 2. That is, exactly what compound is being followed when all the isotope is in microbial biomass? Does the release of ^14^CO_2_ reflect a rate of use of the original (added) compound? Yet, the general method of low concentrations and long incubations has potential for giving a more detailed understanding of how microbes process individual LMW compounds in the soil. One unanswered question that comes from our discussion of possible causes of high concentrations of LMW compounds in soil samples is about the possible effects of disrupting hyphal networks and fine roots. As a result of this disruption, measured concentrations of LMW compounds might increase thereby affecting calculations of turnover rates.

## IS THERE AN IMPORTANT TRANSFER OF DISSOLVED ORGANIC NITROGEN COMPOUNDS FROM AQUATIC AND SOIL ENVIRONMENTS INTO INVERTEBRATES AND PLANTS?

In this review, we have argued that microbes quickly assimilate all available substrates and hold the concentration of amino acids and sugars at very low concentrations. Although the argument is backed by chemical measurement in planktonic aquatic systems, where measured amounts are in the nanomolar range, measured concentrations in soil waters appear to be much higher, in the micromolar range. We argue that the high concentrations were not available to microbes and came from the destruction of soil structure, perhaps the mycorrhizal hyphal network or fine roots, during sample preparation (Hobbie and Hobbie, 2012).

**FIGURE 5:**
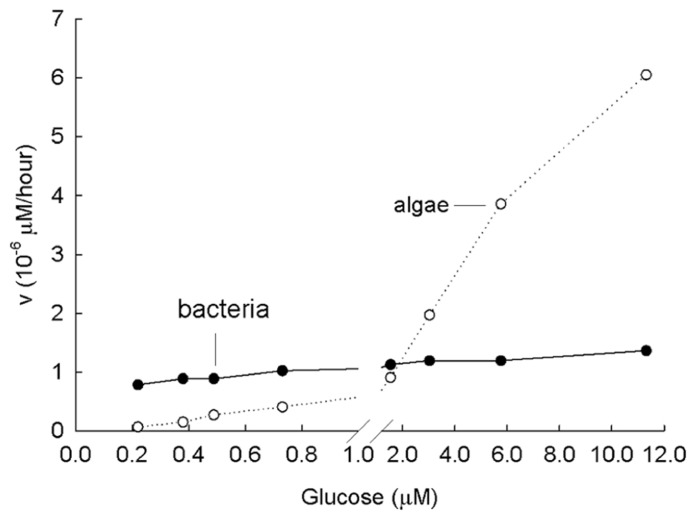
**Laboratory study of Michaelis–Menten kinetics from the incorporation of glucose at a variety of concentrations by a lake bacteria culture (low *K*_**m,**_ low *V*_**max**_) and an algal culture (*Chlamydomonas* sp., high *K*_**m,**_ high *V*_**max**_).** *K*_m_ of bacteria is 27 nM; *K*_m_ of algae is 27,000 nM. Note that uptake of algae resembles diffusion. Modified from Wright and Hobbie (1966).

In the literature, the present understanding is that LMW compounds are present in micromolar concentrations in the soil. What happens in experiments when these high concentrations of labeled amino acids or sugars are added and when plants or animals are present? Fundamental information on the topic comes from studies in aquatic systems and with aquatic organisms. For example, the kinetics of the uptake of glucose in freshwaters was investigated (**Figure 5**) with a bacterial culture (*K*_m_ of 27 nM) and an algal culture of *Chlamydomonas *sp. (*K*_m_ of 27 μM) over a range of low concentrations (Wright and Hobbie, 1966). The bacterial culture was freshly isolated; the algae grew either in the light or on high concentrations of glucose in the dark (Bennett and Hobbie, 1972). In the experiment in **Figure 5**, at low concentrations (<1 μM), the bacterial uptake rises to *V*_max_ as the transport systems become saturated. Over the range 0.3 to 11 μM glucose (0.05–2 mg l^-^^1^) algal uptake increased linearly. This linear increase over the entire range of expected glucose concentrations indicates that a diffusion-like process is driving the uptake. Therefore, if the labeled glucose is added at only one relatively high concentration, which is typical of most soil measurements made, there is no recognition of the importance of concentration added and algal uptake is believed to outcompete bacterial uptake. However, the ecological question is not whether LMW compounds enter the cell but rather whether the contribution of sugars and amino acids is important to the energy and growth requirement of these cells? The value of studying uptake at a number of concentrations of the added substrate is obvious.

Research on the uptake of organic substance into marine invertebrates (Gomme, 2001) has gone through several cycles since it began in the 1870s. Over time, methods have finally improved enough that it is recognized that inshore waters hold a total of 0.1–1 μM free amino acids and that a net uptake of amino acids and glucose by the integument of soft-bodied marine invertebrates does occur at concentrations of ~1 μM (Stephens, 1988). Influx across epidermal membranes was found to be saturable and occurred by means of several substrate-specific pathways (Gomme, 2001). Today, energy budget studies have concluded that dissolved organic matter in the coastal ocean is at least a supplementary energy source for marine invertebrates. However, we note that this conclusion holds only for coastal regions with the highest measured concentrations of amino acids (**Table 1**).

**FIGURE 6:**
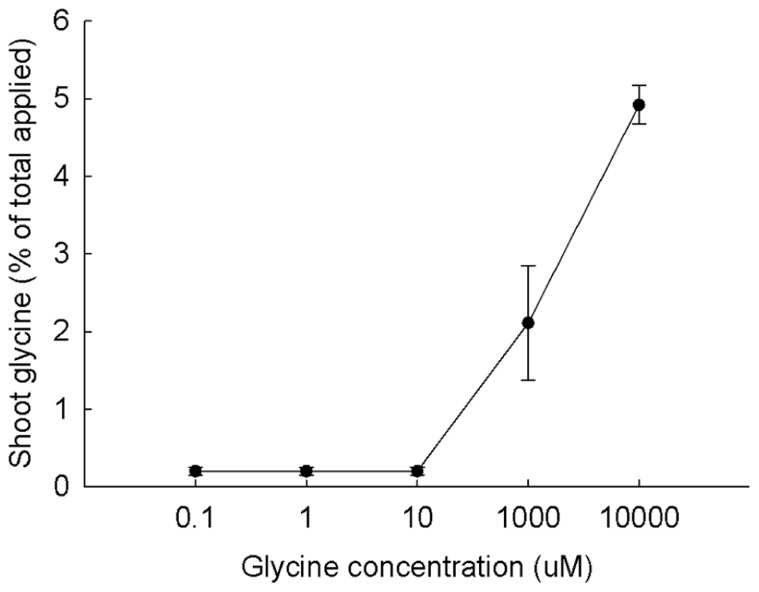
**Uptake of ^**14**^C-glycine at five concentrations from rhizosphere by maize plants.** Plant values are means ± SEM (*n* = 5). From Jones et al. (2005b) with permission.

The evidence presented about the fate of LMW compounds in aquatic systems shows that sugars and amino acids commonly pass through cell membranes and enter eukaryotes both via diffusion and by transport systems. What is the evidence for effect of concentrations of LMW compounds on uptake into plant roots? The only published study we know of is that for glycine uptake in maize plants (Jones et al., 2005b; **Figure 6**). Uptake was very low until the glycine concentration exceeded 10 μM. In a similar study, Campbell (2010) grew cucumber plants on sterile sand and Hoagland’s solution and tested the uptake of ^14^C-leucine into roots at 0.1, 1, 10, 100, and 150 μM. Incorporation was very low until leucine concentrations of 100 and 150 μM were reached. Significant uptake was also measured for uptake of labeled amino acids into tomato roots at concentrations of 10–20 μM (Ge et al., 2009). It is likely that labeled amino acids will enter the roots of many species of plants when uptake is measured at tens and hundreds of micromolar concentrations of substrate.

We are left with uncertainty about uptake rates at the concentrations of amino acids and sugars that are actually available to plant roots. One problem in this type of experiment has already been described because the soil microbes may take up all the added substrate. For example, in a 24 h experiment, labeled amino acids at 100 μM were added to a microcosm with both microbes and wheat roots present; only 6% of the label ended up in the plant roots and microbes removed the rest (Owen and Jones, 2001). In a direct injection of ^15^N-glycine into an arctic soil, after 1 day up to 80% of the ^15^N was found in microbes and 2% in tree roots (Sorensen et al., 2008). The percent in the tree roots was unchanged a year later. Virtually the same results were found when ^15^N-glycine was added to a deciduous forest soil; after 45 min only 0.07% of the added ^15^N was in fine roots and 46% in microbial biomass (McFarland et al., 2002). The percentage in fine roots increased steadily with time, reaching 1.6% after 2 weeks of incubation.

Experimental additions of isotopically labeled amino acids at the same high concentrations that amino acids are chemically measured in the soil have led to the conclusion that plants take up significant amounts of amino acids from soil (Neff et al., 2003). Yet, the ecological significance of organic N uptake for plant N nutrition is still a matter of discussion (Näsholm et al., 2009). As we have discussed, one overlooked topic in almost all publications considering the importance of direct plant uptake of organic N is the ability of soil microbes to remove LMW compounds from soil solution. The typical experiment with a single relatively high concentration of added amino acid and no time course of transfer leaves many questions about rapidity of the removal and of the actual concentrations available to the roots. Once the question about the actual uptake rate is solved, then the larger question can be approached: is the organic nitrogen entering roots from the soil pool an important part of the total nitrogen budget of the plant?

## CONCLUSION

(1) Microbes in natural ecosystems, such as lakes, oceans, and soils, have a starving-survival life style of dormancy, and low activity yet are able to quickly respond to added substrate. While bacteria in planktonic systems keep sugars and amino acids at the nanomolar level, in soil the concentrations of these compounds are measured at the micromolar level. Another difference among ecosystems is that soils have several orders of magnitude more bacteria per unit of volume than do aquatic systems. Fungal hyphae are also abundant in soils and not in the plankton. This disparity of LMW compound concentration and of biomass leads to the conclusion that the high concentrations of LMW compounds in soil are not available to microbes.
(2) The high concentrations of LMW compounds in soil are most likely caused by sampling-induced release of LMW compounds from disturbed soil structures and from damaged roots and mycorrhizal hyphae. The small size of the hyphae and of the fine roots make it very difficult to sample any volume of soil without introducing artifacts.
(3) Kinetic analysis of uptake and turnover of LMW compounds is carried out by isotope dilution in planktonic systems. In soils, however, all added substrate is immediately taken up so dilution analysis is not possible. Instead the biphasic rate of production of ^14^CO_2_ over time has been used to estimate the half-lives of the labeled compounds. Most of the labeled compound is rapidly respired (phase 1) and the percent respired and the rate follows metabolic rules that apply to most microbes. Thus the phase 1 results are independent of the environment and cannot be used to measure half-lives of compounds. In contrast, the longer-term and slow phase 2 release is affected by the environment and could be useful in understanding cycling of individual LMW compounds.
(4) It is possible that the effect of the increased concentrations of LMW compounds caused by sampling disturbance is not confined to carbon cycling methods. Are measures of the rates of *in situ* nitrogen turnover also too high?
(5) Dissolved organic compounds move into cells of plants, fungi, and larval animals by diffusion as well as by various transport mechanisms. If the experimental concentrations of labeled amino acids or sugars greatly exceed natural concentrations, then measured rates of dissolved organic compound use will be higher than the natural rates. Therefore, the effects of different concentrations of substrates used in experiments must be measured. The importance of dissolved organic matter in the carbon and nitrogen budgets of algae, fungi, plants, and even larval animals is still under discussion.
(6) Field experiments where labeled amino acids and sugars are added to soils and the transport of the isotope into trees is measured must also include time-course measures of the rates of uptake into both bacteria and fungi. It is probable that the added substrate or the label passed through microbes before entering trees.

## Conflict of Interest Statement

The authors declare that the research was conducted in the absence of any commercial or financial relationships that could be construed as a potential conflict of interest.
